# Impact of Air-Drying Temperatures on Drying Kinetics, Physicochemical Properties, and Bioactive Profile of Ginger

**DOI:** 10.3390/foods13071096

**Published:** 2024-04-03

**Authors:** Muhammad Nouman Shaukat, Biagio Fallico, Akmal Nazir

**Affiliations:** 1 Department of Agriculture, Food and Environment, University of Catania, Via S. Sofia 100, 95123 Catania, Italy; nouman.shaukat@phd.unict.it (M.N.S.); biagio.fallico@unict.it (B.F.); 2 Department of Food Science, College of Agriculture and Veterinary Medicine, United Arab Emirates University, Al Ain P.O. Box 15551, United Arab Emirates

**Keywords:** ginger, drying kinetics, quality, bioactives, phenols, antioxidants

## Abstract

Ginger (*Zingiber officinale* Roscoe) is a perishable commodity that requires proper processing to maintain its bioactivity. This study evaluated the effect of different air-drying temperatures (50 °C, 60 °C, and 70 °C) on ginger’s drying kinetics and quality attributes. For an enhanced understanding of the drying kinetics, we employed a detailed approach by combining an existing drying model (namely, Midilli) with the Arrhenius model. This combined model facilitates a thorough analysis of how temperature and time concurrently affect the moisture ratio, offering more profound insights into the drying mechanism. A higher drying rate was achieved at 70 °C, yet elevated drying temperatures could compromise the quality attributes of ginger slices. Ginger slices dried at 50 °C displayed improved physicochemical properties and less color browning. The evaluation of the bioactivity profile of resultant ginger extracts also revealed higher total phenolic contents (1875.87 ± 31.40 mg GAE/100 g) and DPPH radical scavenging activity (18.2 ± 0.9 mg TE/kg) in 50 °C treated ginger samples. Meanwhile, the hydroethanolic mixture (70% ethanol) was also reorganized with better extraction efficiency than water and MWF (a ternary blend of methanol, water, and formic acid) solution. The promising outcomes of this study endorse the influence of drying temperature on the quality characteristics and bioactive profile of ginger and the selection of suitable extraction solvents to acquire phenolic-rich extract.

## 1. Introduction

Numerous plant-derived food ingredients have gained considerable importance and research focus due to their biological activities and advantageous characteristics [1,2]. Ginger (*Zingiber officinale* Roscoe), a rhizome from an herbaceous plant, has also gained attention due to its culinary, therapeutic, and biological value. Ginger has been employed since ancient times as an herb, spice, and flavoring agent, concurrently in conventional Chinese medicine. It has been applied to treat various disorders, including flu, asthma, nausea, indigestion, and gastrointestinal distress [3,4]. The nutraceutical and therapeutic potential of ginger is due to the presence of various bioactive and functional substances. The profound bioactive compounds in ginger are gingerols and shogaols, and both these bioactive components, in combination with another functional compound, zingerone, are responsible for the specific aroma of ginger [1,5]. Ginger phytochemicals are a splendid source of phenols and antioxidants; consequently, ginger is also recognized with numerous favorable and health-beneficial properties such as antioxidant activity, antimicrobial activity, anti-inflammatory property, anticarcinogenic capacity, antiemetic activity, neuroprotective potential, cardiovascular protection, hypoglycemic activity [6,7,8].

Consumers have become quite conscious about their food intake, and consequently, their preferences are inclined toward healthy, nutritious, and functional foods [9,10,11]. Interestingly, ginger is also an excellent ingredient for functional food products due to its numerous beneficial attributes [12]. However, ginger’s high moisture content (85–95%) makes it vulnerable to spoilage and microbial degradation, resulting in significant postharvest losses and a loss of freshness, sensory quality, and nutritional value [13,14]. Drying is an extensively utilized food processing and preservation technique. Many perishable food commodities with high moisture content are often dried to avoid decay, spoilage, and microbial degradation [15,16]. Dried ginger serves to manufacture ginger-based processed foods like candies, soft drinks, ginger spices, pharmaceuticals, and cosmetics [17,18,19]. 

Drying is a complicated thermal operation that entails constant transfer of heat and mass during this whole process. The drying process depends on several factors, including the drying temperature, mass flow rate, geometrical features of the dryer, and product [20]. The drying method and temperature can severely modify aromatic and herbal plants’ physicochemical and organoleptic characteristics [21]. The traditional sun-drying technique has been widely used since ancient times. However, its dependence on weather, susceptibility to dust contamination, pest infestations, and microbial deterioration make it less desirable. Therefore, to avoid these drawbacks, mechanical dryers are preferred to obtain superior-quality products [22]. Mechanical drying technologies are also acknowledged for their higher drying rates and energy efficiencies compared to various contemporary drying approaches such as air drying, infrared, microwave, and freeze-drying. For instance, the air-drying technique was reported as a better drying method for the retention of volatile compounds of a product [23]. In addition, hot-air drying was also found to be very competent for maximal color conservation and rehydration ratio of the product. Extensive research has reported and demonstrated that temperature profoundly affects the quality, nutritional content, and bioactive composition of food products [24]. Likewise, in the case of ginger, gingerols are thermally labile and transformed into shogaols during the drying or dehydration process [7,12]. Thus, optimized drying conditions, particularly drying method and temperature, are essential to preserve the bioactivity and quality of dried end product. There are numerous research investigations on the comparison of different drying techniques and technologies for ginger drying [4,13,14,25,26,27]. However, a dynamic study on the effect of different drying temperatures on ginger’s drying kinetics and bioactivity is lacking. Hence, the present work is designed to investigate the influence of different drying temperatures on the drying kinetics, bioactive profile, quality, color, and physicochemical characteristics of ginger slices. 

After the drying and processing of ginger, the subsequent step is acquiring bioactive compounds through a suitable extraction process. An herbal extract’s quality and quantity depend on the food matrix, extraction method, and extraction solvent [28]. In solid–liquid extraction or maceration, solvent selection is the decisive parameter in obtaining a high-quality extract. The selectivity and polarity of the solvent have an explicit impact on the solutes, compounds of interest, and the synergistic relationship between solute and solvent, leading to extraction efficiency [29]. Different extraction solvents have also been employed to extract bioactive compounds and reported as superlative performers compared to a single solvent to improve extraction efficacy. For instance, the combination of acetone with acetic acid and aqueous mixtures of ethanol and methanol, in contrast to pure acetone, ethanol, and methanol, were identified with a higher phenolic value when engaged for the extraction of functional compounds from strawberries [30] and *Pinus densiflora* bark [31], respectively. Various studies have evaluated the efficacy of different solvents, such as methanol, ethanol, acetone, water, etc., and described their qualitative and quantitative influence on ginger’s flavonoids and phenolic contents [32,33,34]. To our understanding, the competency of aqueous blends of different solvents against ginger has not been evaluated. Therefore, the current study is also formulated to determine the impact of different aqueous mixtures of extraction solvents on the extraction efficiency and retrieval of bioactive compounds from ginger. 

## 2. Materials and Methods

### 2.1. The Procurement and Preparation of Sample

Fresh ginger rhizomes were purchased from a local supermarket in Catania (Italy) and then transferred to the food technology laboratory at the Department of Agriculture, Food and Environment, University of Catania (Italy). Ginger rhizomes were washed thoroughly under tap water, manually peeled off using a kitchen peeler, and uniformly sliced into thin slices of around 3 ± 0.2 mm thickness using an electric slicer (ALA-2000, Milan, Italy).

### 2.2. Drying of Ginger

The homogenously cut ginger slices were divided into three equal portions and subjected to hot air drying in a food air dryer (Silvercrest, Hamburg, Germany) at three different drying temperatures (50 °C, 60 °C, and 70 °C). During drying, the moisture content of ginger slices was measured using a thermobalance (Eurotherm, Gibertini, Novate Milanese, Italy) after different time intervals. The drying process continued until the moisture level became almost constant. Then, the drying kinetic parameters, such as drying rate and moisture ratio, were calculated as described by Khan et al. [35]. The drying rate (DR) of ginger slices against each temperature was calculated using the following equation:

(1) $$DR=\frac{M_{t}\;–M_{t+dt}}{dt}$$

where *M_t_* and *M_t+dt_* are the moisture contents of ginger at time *t* and *t+dt*, respectively. Furthermore, the moisture ratio (MR) was calculated using the following equation:

(2) $$MR=\frac{M_{t}-M_{e}}{M_{0}-M_{e}}$$

where *M_t_*, *M*_0_, and *M_e_* are the moisture contents of ginger slices at the time *t*, 0, and at the equilibrium point. The moisture ratio experimental data were also compared with the drying models presented in Table 1 to evaluate the drying kinetics of ginger slices at three different drying temperatures. 

The fitness of experimental data to the drying models was evaluated by calculating the coefficient of determination (R^2^), root mean square error (RMSE), and chi-square (χ^2^) using the following equations:

(3) $$R^{2}=1-\frac{\Sigma {\left(y_{exp.i}-y_{pred.i}\;\right)}^{2}}{\Sigma {\left(y_{exp.i}-\bar{y}\;\right)}^{2}}$$

(4) $$RMSE={\left[\frac{1}{N}\Sigma_{i=1}^{N}\;\;{\left(y_{pred.i}-y_{exp.i}\;\right)}^{2}\right]}^{1/2}\;\;$$

(5) $$\chi^{2}=\frac{\;\Sigma_{i=1}^{N}\;\;{\left(y_{exp.i}-y_{pred.i}\right)}^{2}}{N-z\;}$$

where *y_exp.i_* and *y_pred.i_* are the experimental and predicted values of moisture ratio at *i*th observation, respectively, while *ȳ* is the mean value of experimental moisture ratio; *N* is the number of observations, and *z* is the number of constants in the corresponding model. The non-linear least squares fitting was conducted utilizing the “nlsLM” function from the “minpack.lm” package in R (version 4.2.3). Additionally, R was utilized to generate goodness of fit and diagnostic plots. 

### 2.3. Color Analysis

During the drying process, the digital color pictures of ginger slices were taken after each time interval using a smartphone camera (Oppo Reno 4z, BBK Electronics Co., Shenzhen, China). The images were taken under standardized conditions and parameters to ensure uniformity. These images were subjected to the ImageJ software (version 1.54g), and the obtained responses were utilized to calculate the L* (lightness), a* (redness), and b* (yellowness) color parameters [39]. 

The browning index (BI) of ginger was also determined using the following formula, reported by Zeng et al. [40]:

(6) $$BI=\frac{100\;(x-0.31)}{0.17}$$

where

(7) $$x=\frac{a^{*}+1.75L^{*}\;}{5.645L^{*}+a^{*}-3.012b^{*}}$$

### 2.4. Physicochemical Characteristics of Ginger Powder

After the completion of the drying process, the dried ginger slices were retrieved from the air dryer and allowed to equilibrate at the ambient temperature inside a desiccator for a few hours. The dried ginger was pulverized into ginger powder using a domestic scale kitchen grinder. The ginger powders obtained against three different temperature treatments were subjected to physicochemical characterization, as described in the following sections.

#### 2.4.1. Particle Size Distribution

The particle size distribution of ginger powders was analyzed through a laser diffraction particle size analyzer (Mastersizer 3000, Malvern Panalytical Ltd., Malvern, UK). For the preparation of SOP, the particles were considered opaque (Fraunhofer approximation) and had a density of 0.57 g/cm^3^. The minimum and maximum obscuration levels were set at 0.1% and 15%, respectively. An air pressure of 4 barg, and a feed rate of 50% were set for the sample dispersion. A measurement cycle consisted of three repetitions of 10 s each, and then an averaged particle size distribution was determined. 

#### 2.4.2. Flowability and Compressibility

The bulk density (g/cm^3^) of ginger powders was determined by pouring 2 g of samples into a 10 mL graduated cylinder. In contrast, tap density (g/cm^3^) was measured by tapping the cylinder until a constant volume was achieved. Then, both types of densities were utilized to calculate the flowability and compressibility of the samples. 

The Hausner ratio (flowability) was calculated as per the following formula:

(8) $$Hausner\;Ratio=\frac{Tapped\;density}{Bulk\;density}$$

The Carr index (compressibility of powder) was determined by applying the following formula:

(9) $$Carr\;Index=\left(\frac{Tapped\;density-Bulk\;density}{Bulk\;density}\right)\times 100$$

#### 2.4.3. Solubility

The solubility of ginger powders was measured by dissolving 2 g of ginger powder in 50 mL of distilled water through stirring for 10 min. Then, the resultant solution was centrifuged at 5000 rpm for 10 min. The supernatant was collected and oven-dried at 105 °C for 12 h. The water solubility index in percentage was calculated as follows [41]:

(10) $$Solubility\left(\%\right)=\left(\frac{Weight\;of\;dried\;supernatant}{Sample\;Weight}\right)\times 100$$

#### 2.4.4. Hygroscopicity

The hygroscopicity of ginger powders, referring to the degree to which a solid material absorbs or adsorbs moisture from the ambient environment, was measured through the method described by Hasan et al. [42]. The ginger powder samples (1 g each) were placed on small plastic trays and kept inside a desiccator filled with a saturated NaCl solution (relative humidity: ~76%) for one week at room temperature. After storage, the weight of the samples was measured, and the hygroscopicity was expressed as the weight (g) of adsorbed moisture per 100 g of ginger powder. 

### 2.5. Extraction of Bioactive Compounds

The bioactive compounds from ginger powder were extracted through the maceration process. The extraction efficiency of three different extraction solvents was evaluated, i.e., 70% ethanol, MWF solution (methanol/water/formic acid = 80:19:1), or water [43,44]. Briefly, 5 g of ginger powder was mixed with 100 mL of extraction solvent and left to stir overnight. The resultant solution was centrifuged at 10,000 rpm for 15 min, and the supernatant was filtered through a syringe filter of 0.45 μm to obtain a clear ginger extract. Ginger extract was further analyzed for its total phenolic contents and antioxidative activity, as described below. 

#### 2.5.1. Total Phenolic Contents

The ginger extracts’ total phenolic contents (TPCs) were calculated through the Folin Ciocalteau method reported by Shaukat et al. [45] with minor modifications. Concisely, each sample’s ginger extract (250 μL) was mixed with 1.25 mL Folin Cioaclteau reagent (Carlo Erba Reagents, Italy) and 12.5 mL of distilled water and allowed to react for 3 min. Then, 2.5 mL of 20% sodium carbonate solution was added to each sample solution, and the ultimate volume of 25 mL was made up using distilled water. The comparable blank was also prepared by replacing the ginger extract with a relevant extraction solvent. The resultant sample mixtures were subjected to 1-h incubation under dark conditions at room temperature. Finally, the absorbance of the sample solutions was determined against the blank at 725 nm using a PerkinElmer lambda 25 UV–Vis spectrometer (PerkinElmer, Hopkinton, MA, USA). The absorbance values were plotted against the standard curve of gallic acid, and the TPCs were determined and expressed as milligrams of gallic acid equivalent (GAE) per 100 g of dried sample.

#### 2.5.2. DPPH Antioxidant Assay

The antioxidant activity of ginger extract was evaluated by DPPH radical scavenging assay following the method described by Brand-Williams et al. [46]. Briefly, the ginger extract and its dilutions (50 μL) were incorporated into 3 mL of methanolic DPPH (2,2-diphenyl-1-picrylhydrazyl) solution (100 μM). The resultant blend was mixed and incubated in dark conditions for 1 h at room temperature. The corresponding blank sample was also fabricated by using the relevant extraction solvents instead of ginger extract. The absorbance of all the samples was recorded at 515 nm through PerkinElmer lambda 25 UV–Vis spectrometer (PerkinElmer, MA, USA). The radical scavenging activity of ginger samples was expressed in mg Trolox equivalent (TE) per kg of dried sample attended against the standard curve of Trolox. 

### 2.6. Statistical Analysis

The data collected from the above analyses conducted in three independent replicates were presented as mean values ± standard deviations. The collected data underwent analysis of variance and Fisher’s test to determine significant differences among samples (*p* < 0.05) using the statistical package software Minitab™ version 20 (Minitab, LLC; State College, PA, USA).

## 3. Results and Discussion

### 3.1. Drying Kinetics

#### 3.1.1. Drying Rate

**Figure 1:**
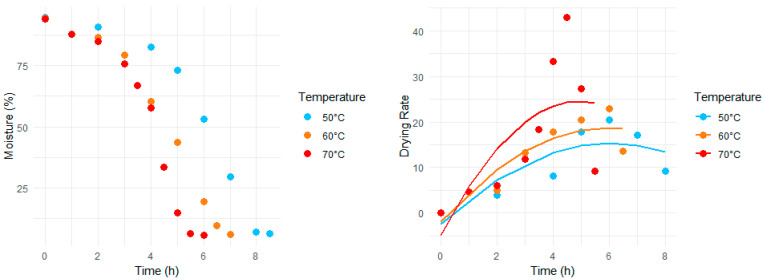
Change in moisture and drying rate of ginger slices dried at different temperatures.

During the drying process, the moisture content of food products declines, although the rate of decrease may vary depending on the applied temperature. Both moisture loss and change in drying rate during the dehydration process at different temperatures are shown in Figure 1. A clear correlation was observed between drying rate and drying temperature over time. The increased mass transfer observed in ginger slices at a relatively higher drying temperature could be attributed to a corresponding enhancement in heat transfer, leading to a rapid dehydration process. The peak drying rate occurred at different time intervals for each drying temperature, with the highest drying rate observed at the maximum drying temperature. Furthermore, the drying rate was higher during the initial stage of the drying process and then dropped with a decline in the sample’s moisture content. This could be attributed to the reduction in the porosity of the sample and subsequent shrinkage, which enhanced the hindrance to water migration inside the sample [37]. If we divide the highest drying rate intervals into the highest drying phase or area under the curve, it would also be evident that the area under the curve is more significant in the case of low-temperature treatment than at higher temperatures. The maximum drying rates for ginger, observed as 23.5 ± 0.81, 24.2 ± 1.37, and 48.2 ± 0.93, were achieved after drying intervals of 7, 6, and 4.5 h, corresponding to temperatures of 50 °C, 60 °C, and 70 °C, respectively. The peak drying rate at 70 °C is not only significantly higher than those observed at the lower temperatures but is nearly double those rates. Furthermore, almost a steep decline in drying rate was observed at the final stage for all three drying temperatures. This decrease in drying rate is associated with the reduction of freely available moisture in the ginger; similar outcomes have already been discussed in the literature [47,48].

The drying rate exhibited a strong correlation with drying time, as an increase in the drying rate resulted in a corresponding reduction in the duration of the drying process. One of the most important variables influencing the quality of drying products is temperature, particularly when it comes to thermosensitive ingredients. Nevertheless, the drying times can be shortened by increasing the drying temperature (as indicated by our results) because it promotes rapid drying.

#### 3.1.2. Moisture Ratio

The moisture ratio is a pivotal metric that quantifies the relative amount of moisture removed from a sample, playing a vital role in depicting drying kinetics. As detailed in Table 1, drying models were applied to the moisture ratios obtained from ginger slices subjected to drying at three distinct temperatures. The optimal model selection was based on criteria including the highest R^2^ and the lowest values for RMSE and χ^2^, with these statistical metrics presented in Table 2. The analysis revealed that the Page and Midilli models significantly outperformed the Lewis and Henderson–Pabis models in accurately describing the drying kinetics of ginger. Upon further comparison, the Midilli model was narrowly deemed the most suitable fit over the Page model, indicating its superior efficacy in explaining the drying behavior of ginger slices.

**Figure 2:**
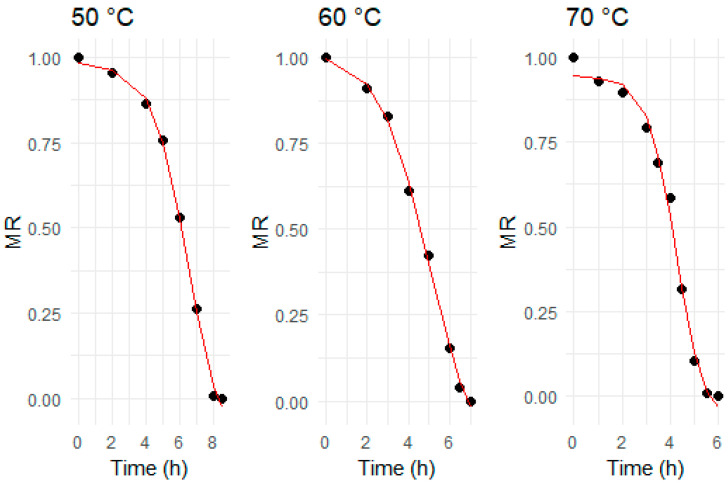
The ginger slices’ experimental moisture ratio (mean values) dried at different temperatures. The red lines represent the best-fit (i.e., Midilli) model.

Figure 2 presents the moisture ratio versus the three different drying temperatures. Consistent with our previous discussion (Section 3.1.2), the moisture ratio in ginger slices showed a swift decrease during high-temperature drying, leading to a quicker drying process compared to lower temperatures. The moisture ratio is intricately linked to diffusivity, which, per Fick’s second law of diffusion, is influenced by moisture movement within the food matrix. The efficiency of diffusivity is also temperature-dependent, exhibiting higher rates in the initial stages of drying due to the increasing temperature of the product [20]. Conversely, effective diffusivity decreases during the final phases of drying due to reduced free water diffusion and accessibility. These results also agree with the literature on black ginger [20] and Urtica dioica leaves [49]. Additionally, Figure 2 compares the predicted MR values against the experimental MR values using the Midilli model, which was the best fit. This comparison further validates the accuracy of the Midilli model at each drying temperature. 

In advancing our modeling of moisture ratio, we integrated temperature effects into the Midilli model by employing the Arrhenius equation, commonly used to characterize the impact of temperature on various food reactions [50]. The Arrhenius equation elucidates the relationship between the reaction rate (in this context, the drying rate) and temperature as follows:

(11) $$k=k_{0}\;exp\left(-\frac{E_{a}}{RT}\right)$$

where *k* is the rate constant, *k*_0_ is the pre-exponential factor, *E_a_* is the activation energy, *R* is the universal gas constant (8.314 J/mol K), and *T* is the absolute temperature in Kelvin. The rate constant *k* in the Midilli model was replaced with the expression from the Arrhenius equation, as follows:

(12) $$MR=a\;exp\left(-k_{0}\exp\left(-\frac{E_{a}}{RT}\right)\;t^{n}\right)+bt$$

**Figure 3:**
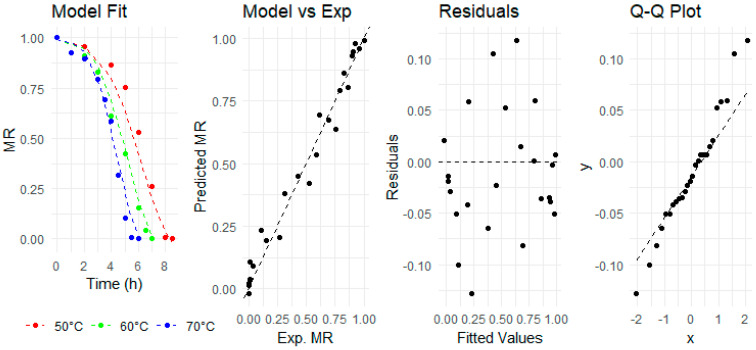
Evaluation of combined model fitness through diagnostics plots for moisture ratio of ginger slices.

This combined equation models the moisture ratio as a function of drying time (*t*) and temperature (*T*), with *k*_0_, *E_a_*, *a*, *n*, and *b* as parameters to be estimated. The combined model was fitted to the experimental data, as shown in Figure 3. 

The combined model’s fitness and compatibility were validated statistically, as illustrated in Table 2, and graphically, via diagnostic plots shown in Figure 3. The R^2^ value for the combined model was slightly less than that of the Midilli model but still was high, approximately 0.94. This demonstrates that the combined model accurately captures the decline in MR across all examined drying temperatures. In the following plot, the experimental MR values also aligned closely to the diagonal line of the combined model’s predicted MR values. The homoscedasticity and linearity of the combined model predictions were also verified in the residuals versus fitted values plot, as the points are evenly scattered without proper alignment or pattern. Finally, the normality of the combined model dataset was also found satisfactory as points closely adhered to the standard line with a little deviation at both ends in the normal quantile–quantile (Q–Q) plot. Conclusively, these diagnostics plots strengthen confidence in the accuracy and predictability of the combined model by providing a satisfactory depiction of the data dynamics. Consequently, it is evident that the combined Midilli–Arrhenius model reliably predicts moisture ratios across various drying temperatures, making it particularly valuable for optimizing ginger drying processes.

### 3.2. Color Analysis

**Figure 4:**
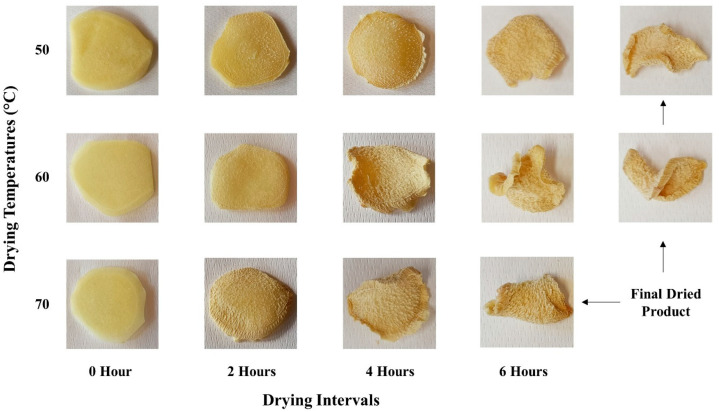
Color of ginger slices during the drying process treated at the different drying temperatures.

**Figure 5:**
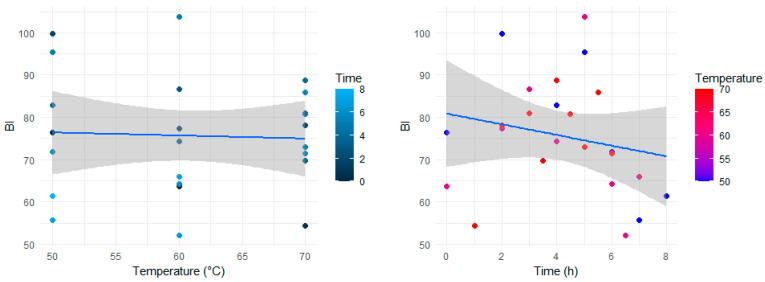
Browning index (BI) of ginger slices dried at different temperatures.

The images of the ginger slices dried at three different temperatures against the coinciding time intervals of the drying process are presented in Figure 4. The color change (and even in texture) that occurred during the drying process can be visualized from these images. The corresponding color parameters (L*, a*, and b*), recorded during the drying process, are detailed in Table 3. The collective influence of all color parameters as a function of temperature and time, represented by the browning index (Equation (6)), is plotted in Figure 5. In comparing all drying temperatures, the highest browning index of 103.70 ± 6.26 was observed for 60 °C after the fifth hour of drying. In contrast, the maximum browning indices were 99.81 ± 2.18 and 88.70 ± 1.08 for 50 °C and 70 °C, respectively. Despite these variations, Figure 5 reveals a lack of a consistent pattern in the browning index across different temperatures and drying intervals. Our analysis indicated a statistically insignificant difference (*p* > 0.05) in the browning index across varying times and temperatures. 

While earlier research [40,51,52] has documented a correlation between the color of dried products and drying temperature, our study did not exhibit significant color changes. Nonetheless, drying at 50 °C demonstrated a slight superiority in our experiments.

### 3.3. Physicochemical Characteristics of Ginger Powder

The physicochemical properties of ginger powder dried at various temperatures are presented in Table 4 and are briefly discussed in this section. Flowability and compressibility are the food ingredients’ crucial features and strongly correlate with the resultant product’s processing efficacy, storage stability, and textural properties [53]. Due to the higher tap density (0.611 ± 0.006 g/mL) of the ginger dried at 50 °C, its flowability (1.72 ± 0.04) and compressibility (42.0 ± 1.30%) were also significantly higher than the other two treatments. Flowability is also associated with particle size distribution, as uniform particle size is characterized by superior flowability features over non-uniform particles. However, the compressibility and flowability of air-dried ginger (against all three temperatures) from the present study are higher than that of sun-dried ginger reported in another investigation [54].

Water solubility is an essential characteristic for efficiently applying a food ingredient. Although there was a non-significant difference in the solubility values, its trend was different from the previously described features, as the ginger powder dried at 70 °C was discovered to be the highest. In contrast, 50 °C treated ginger powder was the lowest. An investigation regarding the manufacturing of date powder through spray drying also proclaimed that the drying or inlet temperature does not significantly influence the powder’s solubility [55]. But in the case of hygroscopicity, the previous tendency has paved its way again, where there was no noticeable disparity between ginger powders at 60 °C and 70 °C. However, the hygroscopicity of ginger powder treated at 50 °C was considerably higher (11.72 ± 0.42%) than other treatments above. The better hygroscopicity of 50 °C ginger powder was more likely due to its fine powder particles. Compared to coarse particles, the fine powder particles might absorb more moisture owing to their greater surface exposure, and similar behavior was also articulated in the case of the dried date fruit powder [42]. 

**Figure 6:**
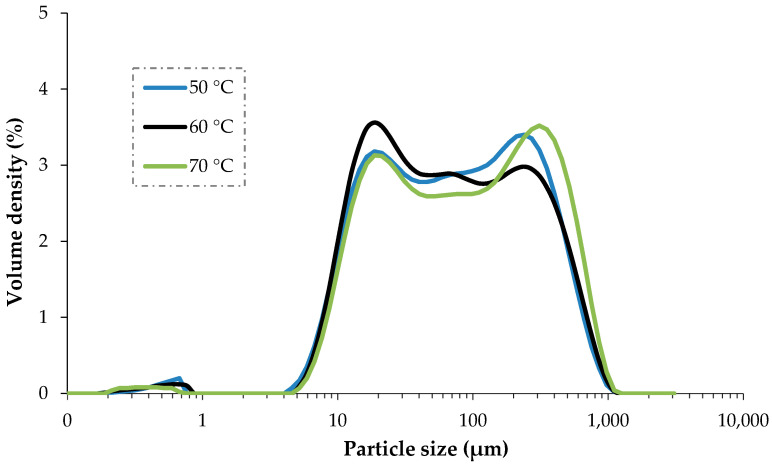
Particle size distribution of ginger powders dried at three different temperatures.

As discussed above, particle size distribution is an essential factor that correlates with other physicochemical properties. From the volume mean diameter D[3,2] parameter of particle distribution (Table 4), it is evident that D[3,2] was increased with increasing the drying temperature and D[3,2] of 50 °C ginger powder is significantly lower than the 70 °C ginger powder while the D[3,2] of sun-dried ginger powder was considerably higher than all of the three treatments of current investigation [54]. Higher drying and evaporation rates trigger rapid moisture removal from the product, promoting hard crust formation in the case of high-temperature treatment. This structural modification at higher temperature employment could also influence the particle size distribution accordingly [56]. The particle size distribution of dried ginger powders against the volume density is also illustrated in Figure 6. It is evident from the graphical representation that the majority of the particle size, against all of the drying temperatures, ranges from 5 μm to 1000 μm, while small peaks were also observed below 1 μm.

### 3.4. Effect of Drying Temperature and Solvents on the Extraction of Bioactive Compounds

**Figure 7:**
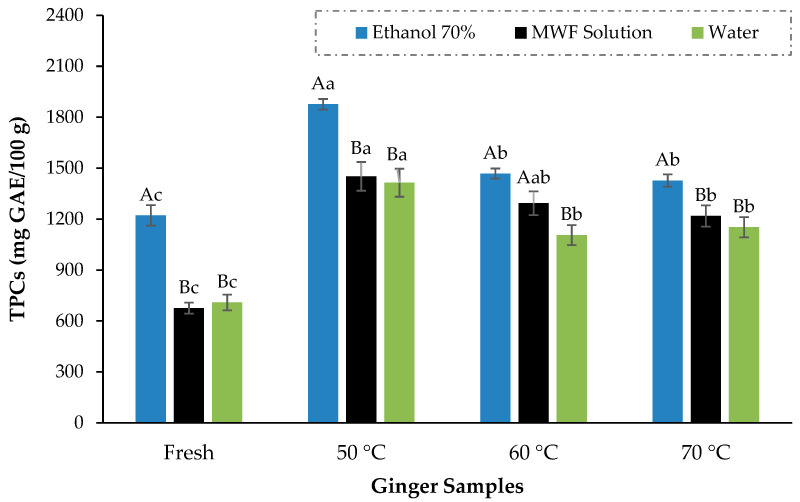
Total phenolic contents (TPCs) of ginger samples dried at different temperatures. The mean values are presented in data bars, with the vertical error bars at each corresponding data bar representing the standard deviation. According to Fisher’s least significant difference test, the data bars with different letters are significantly different (*p* < 0.05) Different capital letters represent the difference among solvents while the different small letters among the samples.

**Figure 8:**
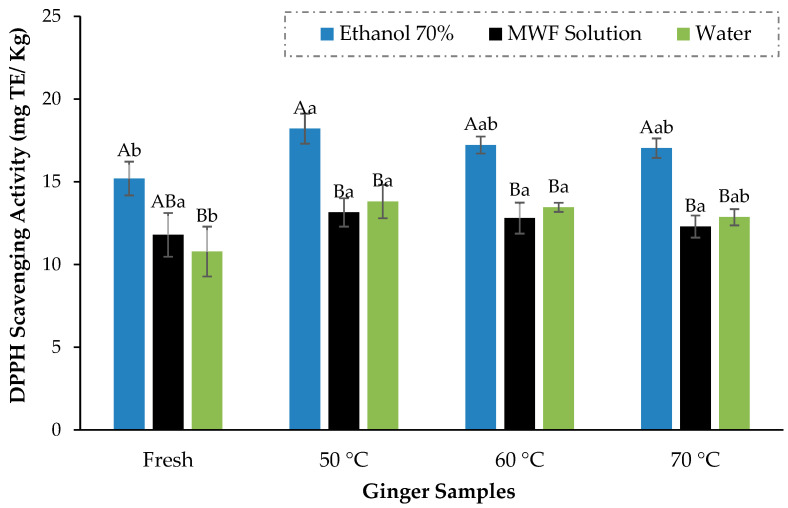
DPPH radical scavenging activity of ginger samples dried at different temperatures. The mean values are presented in data bars, with the vertical error bars at each corresponding data bar representing the standard deviation. According to Fisher’s least significant difference test, the data bars with different letters are significantly different (*p* < 0.05) Different capital letters represent the difference among solvents while the different small letters among the samples.

TPCs of ginger powder dried at different temperatures and extracted in three different solvents are shown in Figure 7. TPCs varied between 1105.9 ± 59.4 and 1875.9 ± 31.4 mg GAE/100 g; the ginger samples dried at 50 °C performed much better than other ginger samples and came up with the highest polyphenols of 1875.9 ± 31.4 mg GAE/100 g which were substantially greater (*p* < 0.05) than the highest TPCs values of 1468.1 ± 29.5 and 1427.1 ± 36.3 mg GAE/100 g against 60 °C and 70 °C, respectively. Although there were variations in TPCs across different temperature treatments, TPCs for ginger dried at all these temperatures were significantly higher than those of fresh ginger (1221.6 ± 60.5 mg GAE/100 g). Similarly, the antioxidant activity of dried ginger (18.2 ± 0.9 mg TE/kg) was significantly higher than fresh ginger (15.2 ± 1.02 mg TE/kg). The outcomes of Guo et al. [21] and An et al. [17] are also in favor of our findings that dried ginger has higher antioxidant activity (DPPH scavenging activity) than fresh ginger. Among different temperature treatments, the antioxidant activity trend was identical to the TPCs, but scavenging values were not markedly different from each other (Figure 8).

The drying of ginger has markedly improved its bioactivity compared to fresh ginger, which is also supported by several previous studies [20,57,58]. The tissues of a food matrix become more fragile during the drying process, triggering the cell wall to break down during grinding, hence improving the uniformity and dissolution in the extraction process. Drying also enhanced the porosity of the dried samples and consequently promoted the better diffusion of solvent, resulting in higher extraction efficiency [27]. In another experiment conducted by Méndez-Lagunas et al. [24], it has also been reported that the higher temperature treatment could also curtail the bioactive compounds in the dried product as there was more decrease in phenolic contents at higher temperature (60 °C) than lower temperature (50 °C) during drying of strawberries.

Similar findings were also expressed by Muthukumar et al. [20], where air drying temperature of 60 °C was recognized as optimum for the black ginger drying in the 40–80 °C temperature range because the reduction in the bioactivity of black ginger was evident above the optimum temperature. In the meantime, we have also been confronted with some previous works where the polyphenolic value of hot air-dried ginger is less than half of our presented results at optimized temperature [14]. So, this study was quite effective in this respect because, remarkably, improvised bioactivity was obtained more than the previously reported literature due to drying temperature optimization. The air-drying process was also described as a better drying approach concerning volatile retention in dried ginger [23].

Along with temperature, the extraction efficiency is also primarily affected by the selection of the extraction solvent. In a contest between the extraction mentioned above solvents, 70% ethanol has emerged as a better extraction solvent in terms of the extraction of antioxidants and phenolic contents from ginger. For instance, for the ginger sample dried at optimal drying temperature (50 °C), the highest total phenolic contents of 1875.87 ± 31.40 mg GAE/100 g were acquired through 70% ethanol, which is also evidently (*p* < 0.05) greater than the TPC values calculated against MWF (1451.237 ± 84.74 mg GAE/100 g) and aqueous (1413.933 ± 82.56 mg GAE/100 g) ginger extract. Makanjuola and Enujiugha [59] have also discovered a massive increment in the total phenolics and flavonoids of the ethanolic extract of ginger in comparison to the aqueous extract. At the same time, in another experiment, ethanol was also acknowledged as a superior extraction solvent to methanol and acetone for extracting functional compounds from ginger [34]. The efficacy of ethanol as an excellent extraction solvent could be attributed to its polarity and synchronization with the food matrix.

## 4. Conclusions

In this current study, the drying kinetics and influence of drying were evaluated on the quality parameters of ginger against different drying temperatures. Based on the statistical analysis, the Midilli model was designated as the best-fit drying model for ginger drying during the drying kinetic study. Furthermore, the integrated Midilli–Arrhenius model can effectively model the drying process, considering both temperature and time concurrently. Ginger’s quality and physicochemical properties were compromised at 70 °C despite a higher drying rate and less drying time. In comparison, 50 °C was identified as an optimal drying temperature. The ginger extract derived from 50 °C also furnished the highest total phenolic contents and antioxidant activity compared to other treatments. On the other hand, 70% ethanol was identified to have better extraction efficacy in deriving optimal bioactive contents from ginger. The present study’s findings help us understand the drying kinetics and the integration of optimal drying and extraction conditions for the maximum extraction of bioactive compounds from ginger.

## Figures and Tables

**Table 1:** Drying models compared with experimental data of ginger drying.

Models	Equation	References
Newton/Lewis	$MR=exp\left(-kt\right)$	[36]
Handerson and Pabis	$MR=a\;exp\left(-kt\right)$	[37]
Page	$MR=exp\left(-kt^{n}\right)$	[38]
Midilli	$MR=a\;exp\left(-kt^{n}\right)+bt$	[35]

**Table 2:** Statistical analysis for drying models’ conformity to experimental moisture ratio values.

Models	Drying Temp.	R^2^	RMSE	χ^2^
Lewis	50 °C	0.656	0.226	0.058
	60 °C	0.640	0.225	0.058
	70 °C	0.679	0.211	0.050
Handerson and Pabis	50 °C	0.694	0.213	0.061
	60 °C	0.765	0.182	0.044
	70 °C	0.669	0.144	0.026
Page	50 °C	0.993	0.033	0.001
	60 °C	0.991	0.035	0.002
	70 °C	0.984	0.048	0.003
Midilli	50 °C	0.995	0.027	0.001
	60 °C	0.993	0.032	0.002
	70 °C	0.997	0.035	0.002
Midilli + Arrhenius	-	0.943	0.056	0.081

**Table 3:** Color parameters of ginger slices dried at different temperatures against the coinciding time intervals.

Drying Temperatures	Color Parameters	Drying Intervals					
		0 h	2 h	4 h	5 h	6 h	Final
50 °C	L*	70.78 ± 1.27 ^a^	62.5 ± 0.87 ^e^	64.87 ± 1.03 ^d^	66.36 ± 0.09 ^cd^	68.63 ± 0.41 ^ab^	68.28 ± 1.24 ^bc^
	a*	−3.67 ± 0.2 ^d^	0.28 ± 0.48 ^c^	1.37 ± 0.28 ^b^	2.85 ± 0.18 ^a^	1.18 ± 0.12 ^b^	1.41 ± 0.37 ^b^
	b*	40.71 ± 0.54 ^a^	41.33 ± 0.83 ^a^	37.43 ± 0.9 ^b^	41.64 ± 1.93 ^a^	35.84 ± 0.29 ^b^	31.55 ± 2.13 ^c^
60 °C	L*	72.83 ± 0.43 ^a^	64.68 ± 0.24 ^bc^	59.79 ± 2.59 ^c^	48.05 ± 5.00 ^d^	68.77 ± 0.83 ^ab^	66.53 ± 1.76 ^b^
	a*	−4.44 ± 0.01 ^d^	−0.43 ± 0.16 ^c^	2.65 ± 0.47 ^b^	6.85 ± 1.11 ^a^	1.23 ± 0.23 ^bc^	1.83 ± 1.1 ^b^
	b*	37.34 ± 0.52 ^a^	36.26 ± 1.63 ^ab^	31.28 ± 1.14 ^bc^	30.24 ± 0.14 ^c^	33.02 ± 4.31 ^abc^	32.26 ± 2.63 ^abc^
70 °C	L*	73.70 ± 2.38 ^a^	59.90 ± 1.73 ^b^	58.55 ± 1.28 ^b^	58.98 ± 2.22 ^b^	61.67 ± 1.00 ^b^	←
	a*	−5.37 ± 0.62 ^c^	1.21 ± 0.45 ^b^	2.69 ± 0.38 ^ab^	3.63 ± 0.94 ^a^	2.98 ± 1.01 ^a^	←
	b*	34.37 ± 1.34 ^a^	34.04 ± 2.82 ^a^	32.60 ± 4.96 ^a^	33.96 ± 0.35 ^a^	35.83 ± 2.60 ^a^	←

← represents that at the corresponding drying temperature, the final drying interval was already accomplished at the preceding drying interval (6 h). Results are reported as mean ± standard deviation. In each row, values with different superscript letters are statistically different (*p* < 0.05) from each other.

**Table 4:** Physicochemical characteristics of ginger slices dried at different temperatures.

Ginger Powder	Bulk Density (g/mL)	Tap Density (g/mL)	Flowability (Hausner Ratio)	Compressibility (Carr Index; %)	Solubility (%)	Hygroscopicity (%)	Particle Size (µm)	
							Dx (50)	D[3,2]
50 °C	0.354 ± 0.004 ^a^	0.611 ± 0.006 ^a^	1.72 ± 0.04 ^a^	42.0 ± 1.30 ^a^	28.60 ± 0.21 ^a^	11.72 ± 0.42 ^a^	74.97 ± 3.00 ^b^	21.07 ± 3.29 ^b^
60 °C	0.364 ± 0.009 ^a^	0.576 ± 0.006 ^b^	1.58 ± 0.02 ^b^	36.9 ± 0.92 ^b^	29.45 ± 0.13 ^a^	9.13 ± 0.39 ^b^	68.67 ± 2.23 ^c^	21.93 ± 0.91 ^ab^
70 °C	0.366 ± 0.002 ^a^	0.566 ± 0.008 ^b^	1.55 ± 0.03 ^b^	35.4 ± 1.30 ^b^	30.40 ± 0.10 ^a^	8.85 ± 0.35 ^b^	93.53 ± 3.85 ^a^	24.77 ± 1.55 ^a^

Results are presented as mean ± standard deviation. In each row, values with different superscript letters are statistically different (*p* < 0.05) from each other.

## Data Availability

The authors confirm that the data supporting the findings of this study are available within the article, further inquiries can be directed to the corresponding author.
